# Cholestatic hepatic injury due to a thyroid storm: a case report from a resource limited setting

**DOI:** 10.1186/1756-6614-5-6

**Published:** 2012-07-28

**Authors:** Davis Kibirige, Daniel Sekikubo Kiggundu, Richard Sanya, Edrisa Mutebi

**Affiliations:** 1 Department of Medicine, Makerere University College of Health Sciences and Emergency Medicine unit, Mulago national referral and teaching hospital, Kampala, Uganda; 2 Department of Medicine, Makerere University College of Health Sciences and Endocrine unit, Mulago national referral and teaching hospital, Kampala, Uganda; 3 Department of Medicine, Makerere University College of Health Sciences, P.O.BOX 7062, Kampala, Uganda

**Keywords:** Thyroid storm, Cholestatic hepatic injury, Cholestasis

## Abstract

**Introduction:**

Thyroid storm is an endocrinological emergency caused by an exacerbation of the hyperthyroid state and is characterized by multi organ dysfunction. Liver dysfunction or injury predominantly of a cholestatic type is one of the atypical manifestations of thyroid storm and has been previously described in literature. However, there have been few published case reports among African patients and from resource limited settings.

**Case report:**

We report a case of a 21 year old Ugandan female patient who presented with a thyroid storm due to untreated Graves’ disease complicated by cholestatic hepatic injury, congestive heart failure and acute kidney injury.

**Conclusion:**

This case highlights the varied multi organ dysfunctions seen in a patient with thyroid storm with emphasis on liver injury mainly to increase awareness among clinicians in resource limited settings. Mechanisms of liver injury due to thyroid storm or hyperthyroidism are discussed in the literature review.

## Introduction

Thyroid storm refers to the sudden onset of life-threatening manifestations of hyperthyroidism. Usually, it complicates Graves’ disease but can also occur in association with toxic multinodular goitre [1].

The condition is often evoked by a precipitating factor such as untreated hyperthyroidism, infectious diseases, acute trauma, thyroidal surgery, excessive iodine intake in diet and administration of iodine-containing materials like amiodarone. The clinical picture is predominated mainly by fever, tachycardia or supraventricular arrhythmias, central nervous system symptoms like severe agitation and altered mentation and gastrointestinal symptoms [2].

Hepatic injury as part of the multi organ dysfunction due to thyroid storm has been described in several case reports and in literature ranging from severe liver dysfunction to acute liver failure and resultant death. A cholestatic picture mainly predominates [3-6].

### Case report

A 21 year old female patient, newly diagnosed with Graves’ disease was referred from a peripheral hospital with a two week history of progressive yellowing of the eyes associated with generalised abdominal distension, marked right hypochondrial pain and mild generalised pruritis. Ten days prior to her admission, she developed worsening exertional dyspnea, easy fatigability, bilateral lower limb swelling , palpitations, orthopnea , profound weight loss and generalised muscular weakness and heat intolerance. She had been amenorrheic for 4 months and was not taking any medications. She had no history of smoking or alcohol consumption.

Clinical examination on admission revealed a thin, diaphoretic and mildly agitated lady. Her axillary temperature was 39.2°C. She had jaundice with bilateral pitting lower limb oedema, a fine resting tremor, exophthalmos and eyelid lag. The thyroid gland was palpable, diffusely enlarged and non tender with a bruit. Cardiovascular examination revealed a rapid, large volume and regular pulse of 140 beats/min with an isolated systolic hypertension of 150/70 mmHg. The jugular venous pressure was raised and neck veins engorged. She had a heaving apex beat displaced to the 6^th^ intercostal space anterior axillary line with a gallop and a soft apical systolic murmur. Abdominal and respiratory examinations revealed a tender hepatomegaly with ascites and reduced breathe sounds on the right infra axillary and scapular areas respectively.

A chest radiograph done revealed a cardiomegaly with a right sided pleural effusion. An abdominal Ultra sound scan done showed an enlarged liver with a normal echo texture, ascites, normal biliary ducts and gall bladder. A thyroid ultra sound scan was not done. Electrocardiography showed sinus tachycardia, with a rate of 142 beats/min and left ventricular hypertrophy by voltage criteria. The Echocardiography showed globally dilated cardiac chambers with moderate mitral and tricuspid regurgitation, an elevated ejection fraction of 82% (40-70%) and moderate pulmonary hypertension.

The laboratory investigations done are summarised in Table 1 below.

**Table 1:** Laboratory investigations of the patient

**LABORATORY PARAMETER**	**PATIENT’S VALUE**	**NORMAL RANGE**
1.Total white blood cell count (x10^9^)	8.7	4.0-9.0
2.Absolute Neutrophil count (x10^9^)	6.6	1.7-7.7
3. Haemoglobin level (g/dl)	**8.0**	**12-17**
4.Serum albumin (g/l)	**21.4**	**35-50**
5. Corrected Calcium (mg/dl)	**11.2**	**9-10.6**
6. Alanine transaminase (U/L)	17.9	0-40
7. Aspartate transaminase (U/L)	**48.2**	**0-40**
8.Alkaline Phosphatase (U/L)	**212**	**40-129**
9.Gamma-glutamyl transpeptidase (U/L)	**82.4**	**0-55**
9.Total bilirubin (μmol/l)	**233.2**	**0-40**
10.Direct bilirubin (μmol/l)	**215.75**	**0-3.4**
11.Serum urea (mmol/l)	**12.4**	**2.7-6.4**
12. Serum creatinine (μmol/l)	**120.7**	**44-106**
13. Urea/Creatinine ratio	**56.4**	**< 20**
14. Serum Sodium (mmol/l)	**130**	**135-150**
15. Serum Creatinine kinase (U/L)	**815**	**26-308**
16. Serum lactate dehydrogenase (U/L)	**409**	**135-225**
17. Serum creatinine kinase-MB isoform-CK-MB (U/L)	**100.9 U/L**	**0-25**
18. Random blood sugar (mg/dl)	7.4 mmol/l	3.5-11
19. Urine pregnancy test	Negative.	
20. HIV serology, Hepatitis Bs Ag test, Hepatitis C antibodies	Negative.	

Thyroid function tests revealed a suppressed thyroid stimulating hormone level (TSH) of <0.005 uIUml (0.27–4.2), raised free thyroxine levels of 267.5 nmol/L (normal range: 66–181) and free triiodothyronine of 10 nmol/L (normal range: 1.3-3.1).

A thyroid nuclear scan showed a diffuse homogenous increased uptake of radioactive iodine with no background uptake. Thyroid stimulating hormone receptor antibody, Antithyroglobulin and antithyroid peroxidase antibodies were not done due to financial constraints.

An impression of thyroid storm due to untreated Graves’ disease with cholestatic liver injury, high output congestive heart failure due to thyrotoxic heart disease and acute kidney injury was made. The patient’s Burch-Wartofsky score for thyroid storm was 90 [7].

She was started on oral carbimazole, Prednisolone, Propranolol, Lugol’s iodine, intra venous fluid replacement, oral anti histamines for the pruritis, oral frusemide and Captopril for the heart failure.

The patient was discharged 10 days later with great improvement and on a follow-up assessment 10 weeks later; she was feeling generally well with resolution of the jaundice and clinically euthyroid. A repeat electrocardiography, echocardiography, renal and liver function tests were all normal.

## Discussion

Thyroid storm is a sudden, life-threatening exacerbation of thyrotoxicosis that is associated with multi organ dysfunction and increased mortality if untreated.

Possible mechanisms responsible for progression to thyroid storm include a steady increase of tissue iodothyronine levels, an augmented cellular response to the circulating thyroid hormones and their effect of enhancing cellular expression of adrenoceptors and sensitivity to circulating cathechoamines [7,8].

The case reported illustrates a thyroid storm due to untreated Graves’ disease with mainly cardiac, renal and gastrointestinal dysfunctions in a 21 year old girl. The diagnosis of Graves’ disease was made basing on the prominent eye signs (exophthalmos and eyelid lag) and a suggestive thyroid nuclear scan (diffuse homogenous increased uptake of radioactive iodine with no background uptake).

Excessive dietary iodine intake is one of the recognised trigger factors of thyroid storm and Graves’ disease [1,2]. However, excessive dietary intake of iodine has been documented to be very uncommon in Uganda. It has been reported in only one district in central Uganda [9].

The patient discussed is from an iodine repleted area with adequate iodine intake in the diet.

Causes of liver injury in hyperthyroidism or thyroid storm are multifactorial. A cholestatic type of hepatic injury of centrilobular intrahepatic form predominates as demonstrated in this case. Cholestatic liver injury is mainly depicted by a raised direct hyperbilirubinemia and alkaline phosphatase levels as shown in the patient’s workup. The latter however may signify an increased osteoblastic response to increased thyroid hormone induced bone resorption [10].

Hepatic damage occurring solely due to thyrotoxicosis or hyperthyroidism has also been thought to be due to ischemic injury resulting from a relative decrease in blood flow despite presence of an increased hepatic metabolic activity [10].

Cholestatic liver injury in patients with hyperthyroidism or thyroid storm due to Graves’ disease can also be associated with other pre existing auto immune liver conditions especially primary biliary cirrhosis (PBC) or autoimmune hepatitis. These are seen in up to 10% of patients [11,12].

Other proposed mechanisms of hepatic dysfunction in hyperthyroidism or thyroid storm include hepatic congestion resulting from heart failure due to thyrotoxic heart disease as showed in the case report. This often leads to hepatic abnormalities like acute hepatocellular injury with hyperbilirubinemia and resultant coagulopathy [13,14]. Anti thyroid drugs especially propylthiouracil also cause cholestatic liver injury and the effect is usually dose dependent [15].

However in this patient, we were unable to exclude any intrinsic co-existing auto immune liver conditions due to limitation of resources. The patient declined a liver biopsy.

## Conclusion

It is of paramount importance to recognise liver injury due to a thyroid storm or hyperthyroidism and to promptly diagnose and treat thyroid storm especially in resource limited settings where intensive care units aren’t easily accessible. This is because severe liver impairment and thyroid storm are associated with increased mortality.

## Consent

Written informed consent was obtained from the patient for publication of this case report. A copy of the written consent is available for review by the Editor-in-Chief of this journal.

## Competing interests

The authors declare that they have no competing interests.

## Authors’ contributions

DK participated in the care of the patient in the emergency unit and drafted the manuscript. DSK, RS and EM participated in the care of the patient in the endocrinology unit and critically revised the contents of the manuscript. All authors approved the final draft of the manuscript for submission for publication.
